# On a Convenient Method of Preparing Eusol

**Published:** 1917-11

**Authors:** 


***On a Convenient Method of Preparing Eusol*** (A Report to the Medical Research Committee from the Department of Pathology, Univ. of Edin.). By J. L. SMITH, Professor of Pathology, Edinburgh Univ., James RITCHIE, Professor of Bacteriology, Edinburgh Univ., and Theodore BETTIE, Research Assist. under the Medical Research Committee. Abstracted from the *British Medical Journal*, Sept. 22, 1917.

The authors cite their earlier contribution on this subject (*B. M. J.*, July 24, 1915) as applying chiefly to large quantities frequently renewed. For any quantity desired they now propose the following method of preparation :

" Take 135 cc. of the B. P. liquor calcis chlorinatae; dilute with water to 1 litre, add ten grams of boric acid, and shake up till dissolved. The solution remains clear, and without further treatment is ready for use. If preferred, a saturated solution of boric acid may be stocked at room temperature; this contains 4 o/o boric acid, therefore 250 cc. gives the amount required for 1 litre eusol. In making eusol in this way the 135 cc. of liquor calcis chlorinatae should be diluted to 750 cc. and 250 cc. of boric acid solution added. This prevents the formation of the precipitate which occurs if boric acid be added to undiluted liquor calcis chlorinatae. "

The authors note that this solution may be prepared at a moment's notice.

For intravenous injection in septicemic cases, sodium chloride should be added in the proportion of 8.5 gram to the litre.

The liquor calcis chlorinatae is a 10 o/o solution of bleaching powder in water. Its keeping power is fairly good in spite of the deterioration in the quality of the chloride of lime manufactured during the war. The available chlorine can easily be determined by titration with N/10 sodium arsenite solution. This should be done especially before intravenous injections.

Standard Eusol is a solution of which
1 cc. = 1 cc. N/10 sodium arsenite.
= 0.0035 gram chlorine.
= 0.0026 gram hypochlorus acid.

For the preparation of the sodium arsenite solution, the authors quote Cumming and Kay as follows :

" Weigh out accurately in a small porcelain basin 12.37 grams of pure arsenious oxide (previously dried in a desiccator); add 10 cc. of sodium hydroxide solution (twice normal), and warm the basin on the steam bath, stirring the mixture gently until the oxide dissolves (about five minutes). Transfer the solution and the rinsings of the basin to a 250 c.c. standard flask and add 20 cc. of dilute hydrochloric acid (twice normal); and then add about 8 grams of sodium bicarbonate or 100 cc. (of cold saturated solution) and dilute the solution to the graduation mark. "

The authors describe the application of the test as follows :

" Take 10 cc. of the liquor calcis chlorinatae; dilute to 100 cc., 10 cc. ; of this solution = say 0.74 cc. N/10 sodium arsenite solution, the end point of the reaction being determined by potassium iodide starch solution or paper. From this a simple calculation shows that 135 cc. of liquor calcis chlorinatae per litre is needed to give the standard eusol. When bleaching powder of high quality is used, about 125 cc. will be found sufficient. "

